# Diversity and functions of volatile organic compounds produced by *Streptomyces* from a disease-suppressive soil

**DOI:** 10.3389/fmicb.2015.01081

**Published:** 2015-10-09

**Authors:** Viviane Cordovez, Victor J. Carrion, Desalegn W. Etalo, Roland Mumm, Hua Zhu, Gilles P. van Wezel, Jos M. Raaijmakers

**Affiliations:** ^1^Department of Microbial Ecology, Netherlands Institute of Ecology (NIOO-KNAW)Wageningen, Netherlands; ^2^Laboratory of Phytopathology, Wageningen UniversityWageningen, Netherlands; ^3^Plant Research International, Business Unit Bioscience, Wageningen University and Research CentreWageningen, Netherlands; ^4^Centre for Biosystems GenomicsWageningen, Netherlands; ^5^Molecular Biotechnology, Institute of Biology, Leiden UniversityLeiden, Netherlands

**Keywords:** Actinobacteria, SPME-GC-MS, antifungal activity, plant growth promotion, suppressive soil

## Abstract

In disease-suppressive soils, plants are protected from infections by specific root pathogens due to the antagonistic activities of soil and rhizosphere microorganisms. For most disease-suppressive soils, however, the microorganisms and mechanisms involved in pathogen control are largely unknown. Our recent studies identified Actinobacteria as the most dynamic phylum in a soil suppressive to the fungal root pathogen *Rhizoctonia solani*. Here we isolated and characterized 300 isolates of rhizospheric Actinobacteria from the *Rhizoctonia*-suppressive soil. *Streptomyces* species were the most abundant, representing approximately 70% of the isolates. *Streptomyces* are renowned for the production of an exceptionally large number of secondary metabolites, including volatile organic compounds (VOCs). VOC profiling of 12 representative *Streptomyces* isolates by SPME-GC-MS allowed a more refined phylogenetic delineation of the *Streptomyces* isolates than the sequencing of 16S rRNA and the house-keeping genes *atpD* and *recA* only. VOCs of several *Streptomyces* isolates inhibited hyphal growth of *R. solani* and significantly enhanced plant shoot and root biomass. Coupling of *Streptomyces* VOC profiles with their effects on fungal growth, pointed to VOCs potentially involved in antifungal activity. Subsequent assays with five synthetic analogs of the identified VOCs showed that methyl 2-methylpentanoate, 1,3,5-trichloro-2-methoxy benzene and the VOCs mixture have antifungal activity. In conclusion, our results point to a potential role of VOC-producing *Streptomyces* in disease suppressive soils and show that VOC profiling of rhizospheric *Streptomyces* can be used as a complementary identification tool to construct strain-specific metabolic signatures.

## Introduction

Disease-suppressive soils are soils in which plants are effectively protected from infections by specific root pathogens due to antagonistic activities of soil and rhizosphere (micro)organisms (Hornby, 1983; Weller et al., 2002). This phenomenon has been described worldwide, but the responsible (micro)organisms and underlying mechanisms are largely unknown for most suppressive-soils (Weller et al., 2002; Mendes et al., 2011; Chapelle et al., 2015). In recent studies, we identified the microbiome of a soil suppressive to *Rhizoctonia solani*, an economically important soil-borne fungal pathogen of many crops including sugar beet, potato, and rice (Mendes et al., 2011; Chapelle et al., 2015). PhyloChip-based metagenomics detected more than 33000 bacterial and archaeal taxa in the rhizosphere of sugar beet seedlings grown in the *Rhizoctonia*-suppressive soil and revealed bacterial groups consistently associated with the disease suppressive state. Among the top 10% of most dynamic taxa (i.e., taxa relatively more abundant in suppressive than in non-suppressive soil), Actinobacteria were the most dynamic phylum found in the rhizosphere of sugar beet seedlings growing in the suppressive soil.

Actinobacteria are ubiquitously found in nature and the phylum comprises more than 500 formally described species (Goodfellow, 2012; Labeda et al., 2012). Many Actinobacteria are multicellular bacteria with a complex life cycle and are renowned for the production of an exceptionally large number of bioactive metabolites (Claessen et al., 2014). Members of the genus *Streptomyces* produce over 10000 secondary metabolites, including volatile organic compounds (VOCs) (Bérdy, 2005; Hopwood, 2007; van Wezel et al., 2009). Approximately 1000 microbial VOCs have been identified to date (Piechulla and Degenhardt, 2014). Although the production of VOCs by microorganisms is known for many years (Zoller and Clark, 1921; Stotzky and Schenck, 1976), it is only since the last decade that an increasing number of studies have reported on the diversity and potential functions of these compounds. The blend of VOCs released by microorganisms is diverse and complex. Microbial VOCs belong to different classes of compounds such as alkenes, alcohols, ketones, terpenes, benzenoids, aldehydes, pyrazines, acids, esters, and sulfur-containing compounds (Effmert et al., 2012). The same VOCs can be found for different, often unrelated, microorganisms but some VOCs are unique to specific microorganisms (Schulz and Dickschat, 2007; Garbeva et al., 2014). Microbial VOCs display versatile functions: they inhibit bacterial and fungal growth, promote or inhibit plant growth, trigger plant resistance and attract other micro- and macro-organisms (Ryu et al., 2003, 2004; Vespermann et al., 2007; Kai et al., 2009; Verhulst et al., 2009; Bailly and Weisskopf, 2012; Hagai et al., 2014; Schmidt et al., 2015). Furthermore, VOCs have been proposed to function as signaling molecules in inter- and intra-specific interactions and in cell-to-cell communication. To date, however, the natural functions of microbial VOCs and their modes of action remain largely unknown (Kai et al., 2009; Kim et al., 2012; Schmidt et al., 2015).

Here we studied the diversity and functions of VOCs produced by different *Streptomyces* from the rhizosphere of sugar beet seedlings grown in a *Rhizoctonia*-suppressive soil. We first isolated and characterized 300 Actinobacteria. As *Streptomyces* represented almost 70% of all isolates, subsequent VOC analyses, phylogeny, antifungal activity and plant growth assays were conducted with this group of Actinobacteria. By coupling SPME-GC-MS and hierarchical clustering of VOC profiles, we identified VOCs potentially involved in antifungal activity.

## Materials and methods

### Selective isolation of actinobacteria

Actinobacteria were isolated from the rhizosphere (roots with adhering soil) of sugar beet plants grown in a soil suppressive to *R. solani*. The soil was previously collected in 2003 and 2004 from an agricultural sugar beet field close to the town of Hoeven, the Netherlands (51°35′10″N 4°34″44′E). For the collection of Actinobacteria from the rhizosphere, sugar beet seeds (cultivar Alligator) were sown in square PVC pots containing 250 g of field soil with an initial moisture content of 10% (v/w). Plants were grown in a growth chamber (24°C/24°C day/night temperatures; 180 μmol light m^−2^ s^−1^ at plant level during 16 h/d; 70% relative humidity) and watered weekly with standard Hoagland solution (macronutrients only). After 3 weeks of plant growth, 1 g of sugar beet roots with adhering soil was suspended in 5 mL of potassium-phosphate buffer (pH 7.0). Samples were vortexed and sonicated for 1 min. To enrich for different genera of Actinobacteria, a number of treatments were applied to the soil suspension (Supplementary Table S1). Single colonies were picked based on the morphology and purified on fresh agar plates. Isolates were stored in glycerol (20%, v/v) at −20 and −80°C.

### Characterization of actinobacteria

All 300 Actinobacterial isolates were characterized by 16S rRNA gene sequencing. PCR amplifications were conducted using primers 8F (5′- AGAGTTTGATC CTGGCTCAG - 3′) and 1392R (5′- ACGGGCGGT GTGTACA - 3′) or 27F (5′- GAGTTTGATCCTG GCTCAG - 3′) and 1492R (5′- ACCTTGTTACGACGACTT - 3′) (Lane, 1991; Deangelis et al., 2009). For obtaining DNA, bacterial cells were disrupted by heating at 95°C for 10 min. For spore forming isolates, cells were disrupted in the microwave at 650 W for 30 s in TE buffer. Suspensions were centrifuged at 13000 rpm for 10 min. After centrifugation, 2 μl of the supernatants were used for the PCR reactions. PCR products were purified and sequenced at Macrogen Inc. Isolates were characterized based on sequence identity with 16S rRNA gene sequences in the Greengenes database (McDonald et al., 2012) (http://greengenes.lbl.gov/).

### Coupling *Streptomyces* isolates to OTUs detected by PhyloChip

16S rRNA gene sequences of 173 *Streptomyces* isolates were compared with the 16S rRNA gene sequences of *Streptomyces* OTUs previously identified by PhyloChip-based metagenomic analysis as the top 10% of most abundant taxa associated with disease suppressiveness (Mendes et al., 2011). Phylogenetic analysis was performed with Muscle in MEGA6 (Tamura et al., 2013) and iTOL (Letunic and Bork, 2011) (http://itol.embl.de/). A Neighbor-joining consensus tree (Saitou and Nei, 1987) with 1000 bootstrap replicates (Felsenstein, 1985) was constructed using Tamura-Nei model (Tamura and Nei, 1993) with gamma distribution. A total of 11 isolates, which were closely related to the isolates detected by PhyloChip, was selected to study the composition of emitted VOCs and their *in vitro* effects on fungal and plant growth. *Streptomyces lividans* 1326 (Cruz-Morales et al., 2013) was used as a reference strain.

### Characterization of selected *Streptomyces* isolates

The 11 *Streptomyces* isolates were characterized based on colony morphology and by sequence analysis of the house-keeping genes *recA* (recombinase A) and *atpD* (ATP synthase subunit B). These genes were amplified and sequenced as previously described (Guo et al., 2008). Partial sequences of *recA* (500 bp), *atpD* (423 bp), and 16S rRNA (516 bp) genes of *Streptomyces* were concatenated to yield an alignment of 1439 sites. A concatenated phylogenetic tree supplemented with sequences of *Streptomyces* strains with a sequenced genome (NCBI database) was constructed using UPGMA with the Tamura-3 parameter calculation model with gamma distribution and 1.000 bootstrap replicates. All sequences were deposited to GenBak and have been assigned to accession numbers: KT60032-KT600042 (16S rRNA gene), KT600043-KT600053 (*recA* gene), and KT600054-KT600064 (*atpD* gene).

### Collection and analysis of *Streptomyces* VOCs

For trapping the VOCs, the *Streptomyces* isolates were inoculated individually in 10 ml sterile glass vials containing 2.5 ml of GA medium (Zhang, 1990) with three replicates each. Vials containing medium only served as controls. All vials were closed and incubated at 30°C. After 7 days, VOCs from the headspace of each vial were collected by solid phase microextraction (SPME) with a 65-mm polydimethylsiloxane-divinylbenzene fiber (Supelco, Bellefonte, USA).

*Streptomyces* VOCs were analyzed by GC-MS (Agilent GC7890A with a quadrupole MSD Agilent 5978C). VOCs were thermally desorbed at 250°C by inserting the fiber for 2 min into the hot GC injection port. The compounds released were transferred onto the analytical column (HP-5MS, 30 m × 0.25 mm ID, 0.25 μm—film thickness) in splitless mode. The temperature program of the GC oven started at 45°C (2-min hold) and rose with 10°C min^−1^ to 280°C (3-min hold). Mass scanning was done from 33 to 300 *m/z* with a scan time of 2.8 scans s^−1^. GC-MS raw data were processed by an untargeted metabolomics approach. MetAlign software (Lommen and Kools, 2012) was used to extract and align the mass signals (s/n = 3). MSClust was used to remove signal redundancy per metabolite and to reconstruct compound mass spectra as previously described (Tikunov et al., 2012). VOCs were tentatively annotated by comparing their mass spectra with those of commercial (NIST08) and in-house mass spectral libraries. Linear retention indices (RI) of VOCs were calculated as previously described (Strehmel et al., 2008) and compared with those in the literature. VOCs selected for *in vitro* antifungal assays [methyl butanoate (≥98%), methyl 2-methylpentanoate (≥98%), methyl 3-methylpentanoate (≥97%), 1,3,5-trichloro-2-methoxy benzene (99%) and 3-octanone (≥98%)] were confirmed with authentic reference standards obtained at Sigma-Aldrich. Processed VOC data were log transformed and auto-scaled using the average as an offset and the standard deviation as scale [raw value-average (offset)/SD (scale)]. Log transformed data were then subjected to multivariate statistical analysis. One-way ANOVA was performed with GeneMaths XT Version 2.11 (Applied Maths, Belgium) to identify VOCs significantly different from the control (medium only) [*p* < 0.05; with false discovery rate (FDR) correction]. After that, hierarchical cluster analysis (HCA) using Pearson's correlation coefficient with UPGMA algorithm was performed.

### VOC-mediated antifungal activity

The effect of *Streptomyces* VOCs on the growth of the fungus *R. solani* was investigated using the bottoms of two 90-mm-diameter Petri dishes allowing physical separation between the bacteria and the fungus. One bottom contained a *Streptomyces* isolate on GA medium, previously incubated at 30°C for 4 days. The other bottom contained a plug of *R. solani* mycelium on 1/10th Tryptone Soy Agar (TSA, Oxoid). Both Petri dishes were sealed facing each other and incubated at 25°C with the Petri dish containing the *Streptomyces* on the bottom to avoid spores transferring to the plate with the fungus. As a control, the Petri dish containing *R. solani* was exposed to a Petri dish containing GA medium only. Fungal growth inhibition was calculated by measuring the radial growth of the fungal hyphae after 1, 2, and 3 days of incubation. Percentage of inhibition was calculated as [(diameter of fungus in control − diameter of fungus exposed to VOCs)^*^100/diameter of fungus in control] for each of the 3 replicates. Student's *t*-Test was performed to determine statistically significant differences compared to the control (*p* < 0.05, *n* = 3).

### Antifungal activity of synthetic VOCs

Methyl butanoate (≥98%), methyl 2-methylpentanoate (≥98%), methyl 3-methylpentanoate (≥97%), 1,3,5-trichloro-2-methoxy benzene (99%), and 3-octanone (≥98%) were obtained at Sigma-Aldrich. All VOCs were dissolved in methanol with final concentrations ranging from 1 M to 1 nM (10-fold dilutions). Assays were performed using a standard 90 mm-diameter Petri dish with the fungal plug on 1/10th TSA medium on top and with a sterile paper filter (1.5 × 1.5 cm) on the bottom. Twenty microliters of each VOC dissolved in methanol were applied on the paper filter, plates were immediately sealed and incubated at 25°C. Radial hyphal growth of the fungus was measured after 1 and 2 days of exposure to single or a mixture of the 5 VOCs and compared to control (empty top of a Petri dish). To check whether the solvent itself had any effect on growth of the fungus, *R. solani* was also exposed to methanol alone. Student's *t*-Test was performed to determine statistically significant differences compared to the control (*p* < 0.05, *n* = 3 − 5).

### VOC-mediated plant growth promotion

To determine whether *Streptomyces* VOCs had an effect on plant growth, *Arabidopsis thaliana* seedlings were exposed to the VOCs emitted by the different isolates. *A. thaliana* seeds (wild-type Col-0) were surface sterilized as previously described (van de Mortel et al., 2012) and sown on 90-mm-diameter Petri dishes containing 50 ml of 0.5X Murashige and Skoog medium (Murashige and Skoog, 1962) supplemented with 0.5% (w/v) sucrose. The 90-mm-diameter Petri dishes were placed inside a 145-mm-diameter Petri dish, sealed and incubated in a climate chamber (21°C/21°C day/night temperatures; 180 μmol light m^−2^ s^−1^ at plant level during 16 h/d; 70% relative humidity). After 7 days, 35-mm-diameter Petri dishes containing *Streptomyces* isolates growing on GA medium (previously incubated at 30°C for 1 week) were added to the 145-mm Petri dishes with the *A. thaliana* seedlings. Plates were sealed and kept at 21°C. After 14 days, plant fresh weight was determined. In addition, plant dry weight was measured after drying shoots and roots overnight in an incubator at 65°C. Student's *t*-Test was performed to determine statistically significant differences compared to the control treatment (plants exposed to medium only).

## Results

### Diversity of actinobacteria isolated from suppressive soil

**Figure 1:**
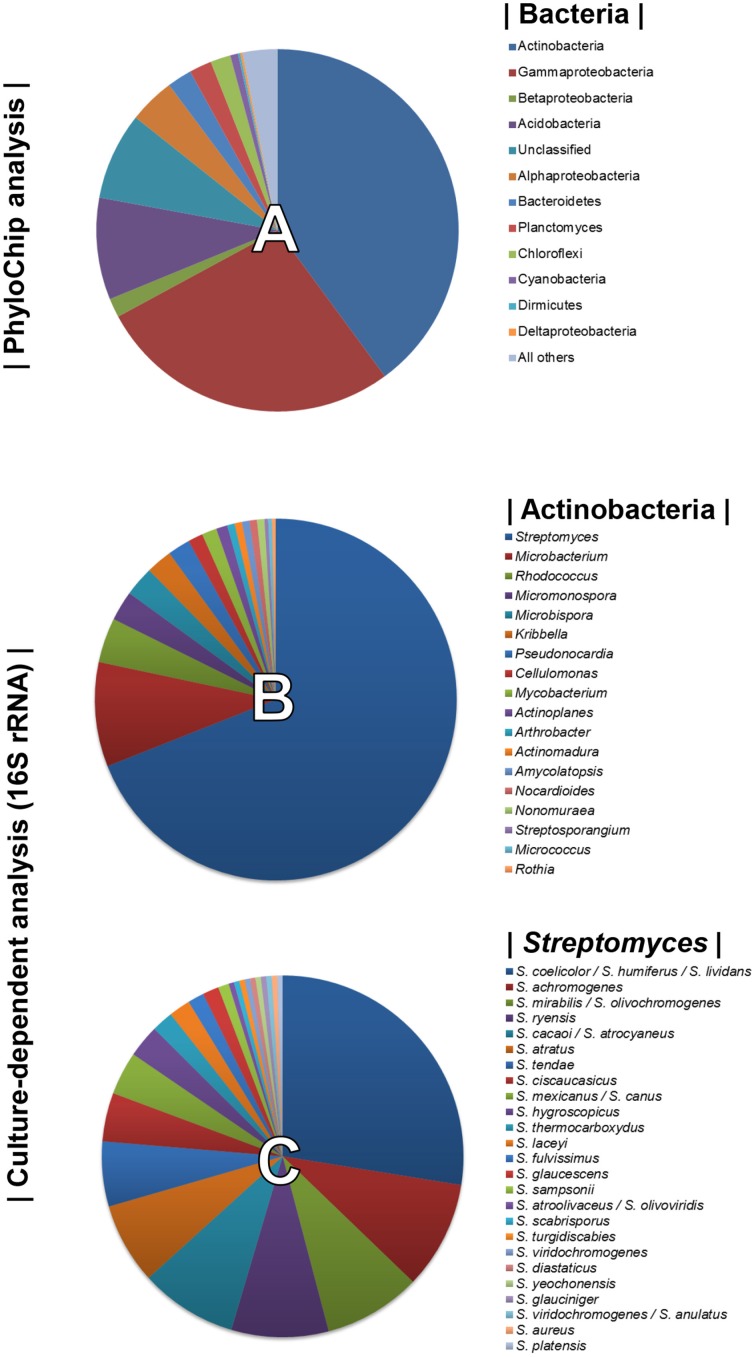
**Top 10% most dynamic bacterial (and archaeal) phyla detected by PhyloChip analysis of the rhizosphere microbiome of sugar beet seedlings grown in ***Rhizoctonia***-suppressive soil (pie chart A, adapted from Mendes et al., 2011)**. Diversity of Actinobacteria (pie chart **B**) and of *Streptomyces* species (pie chart **C**) isolated from the rhizosphere of sugar beet seedlings grown in *Rhizoctonia*-suppressive soil (this study).

Using PhyloChip-based metagenomic analyses, we previously described the diversity of the bacterial community associated with the rhizosphere of sugarbeet plants grown in a *Rhizoctonia*-suppressive soil (Mendes et al., 2011). Actinobacteria were prominently more represented in the suppressive soil than in the non-suppressive (conducive) soil. Bacterial diversity detected by the PhyloChip used in the aforementioned study is displayed in Figure 1A. To select as many Actinobacterial isolates as possible, several pre-treatments of the rhizospheric soil and different selective media were used for their isolation (Supplementary Table S1). A total of 300 Actinobacterial isolates were obtained and characterized by 16S rRNA gene sequencing. Based on the sequence similarities (95–100%) to the 16S rRNA gene sequences available in the Greengenes database (used as reference in the PhyloChip analyses), 18 different genera of Actinobacteria were identified. These were *Streptomyces, Microbacterium, Rhodococcus, Micromonospora, Microbispora, Kribbella, Pseudonocardia, Cellulomonas, Mycobacterium, Actinoplanes, Arthrobacter, Actinomadura, Amycolaptosis, Nocardioides, Nonomureae, Streptosporangium, Micrococcus*, and *Rothia* (Figure 1B). The genus *Streptomyces* was the most abundant, representing 69% of all isolates and at least 25 different species based on 16S rRNA gene sequences (Figure 1C).

### Phylogenetic analysis of *Streptomyces* isolates

**Figure 2:**
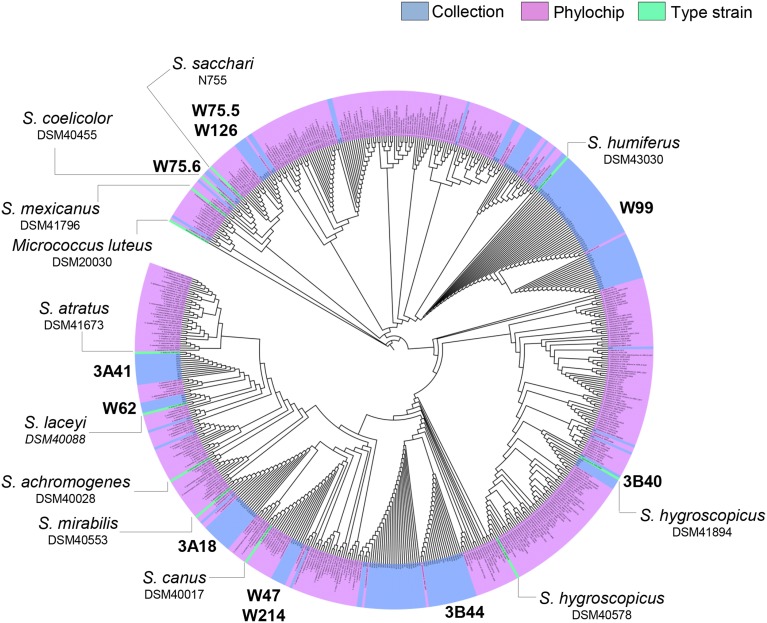
**Neighbor-joining phylogenetic tree based on 16S rRNA gene sequences of the ***Streptomyces*** collection obtained in this study (in blue), ***Streptomyces*** detected by Phylochip analysis (in pink), and ***Streptomyces*** type strains (in green)**. *Streptomyces* isolates selected for VOC analysis are indicated in bold.

**Figure 3:**
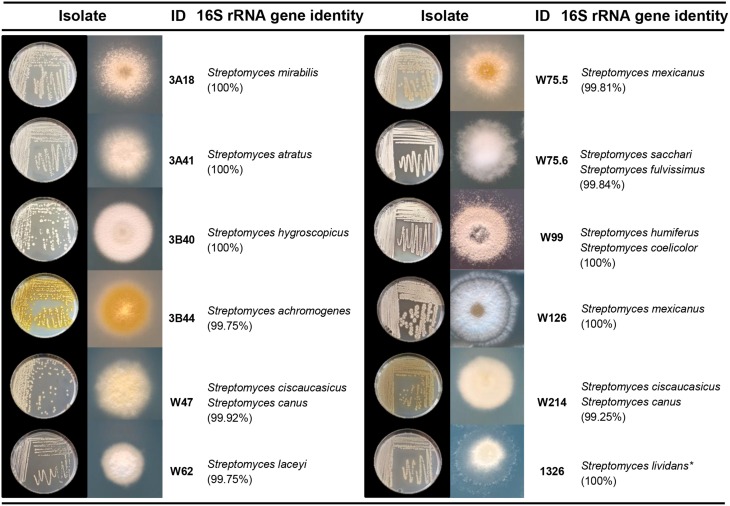
**Characterization of ***Streptomyces*** isolates used in this study**. Species names are based on 16S rRNA gene sequence comparison using the Greengenes database. Pictures depict 4–7 day-old isolates grown on GA medium. ^*^S. lividans 1326 refers to John Innes Center collection number and corresponds to *S. lividans* 66 (Hopwood et al., 1983).

To select *Streptomyces* isolates for VOC and functional analyses, 16S rRNA gene sequences of the *Streptomyces* isolates (*n* = 173) obtained in this study were compared with those of the representative *Streptomyces* OTUs (*n* = 430) originally detected by PhyloChip (Mendes et al., 2011). A phylogenetic tree was constructed using these sequences and the sequences of different *Streptomyces* type strains (Figure 2). This comparison led to the selection of 11 isolates (Figure 3). We then constructed phylogenetic trees with these 11 isolates, their closest type strains, other *Streptomyces* species with sequenced genomes and the reference strain *Streptomyces lividans* 1326 (Supplementary Figure S1A). Additionally, we sequenced the house-keeping genes *atpD* and *recA* (Supplementary Figure S1B). Concatenation of *atpD, recA*, and 16S sequences allowed a better resolution of the different *Streptomyces* isolates than based on 16S sequences only. However, closely related but phenotypically different isolates, like *Streptomyces* strains W75.5 and W126 (Figure 3), could not be distinguished based on these three molecular markers.

### VOC profiling of *Streptomyces* isolates

**Figure 4:**
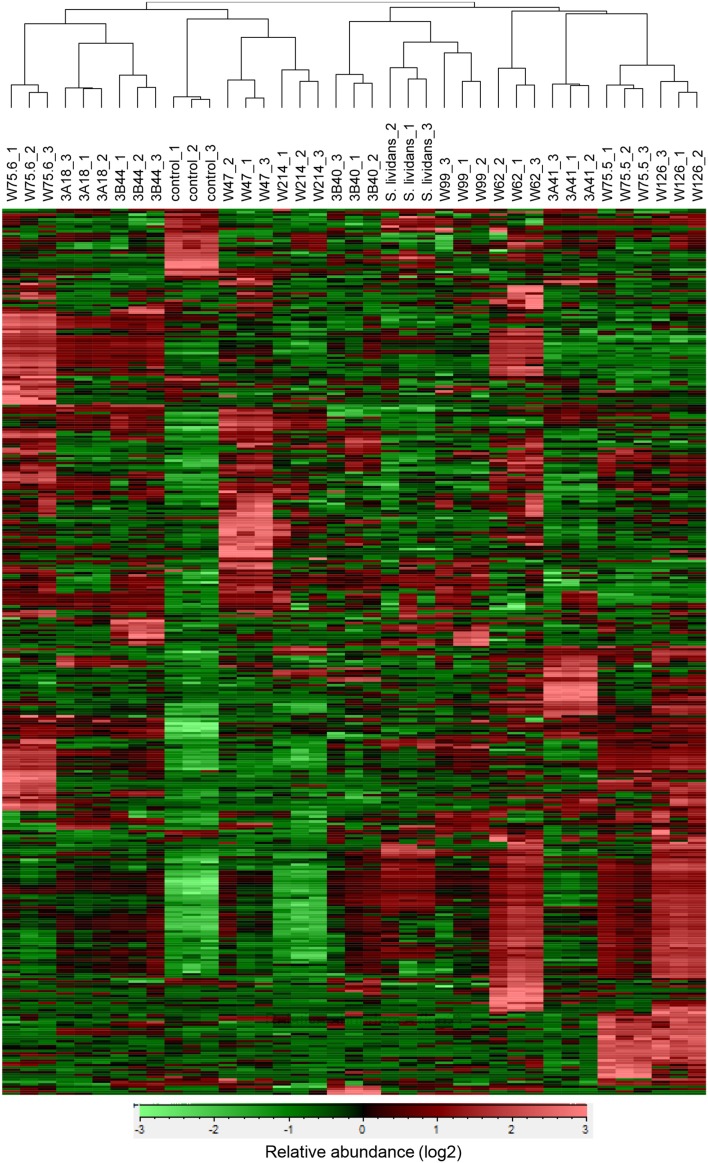
**Hierarchical cluster and heat-map analyses of VOC profiles of the selected ***Streptomyces*** isolates**. Columns represent three replicate VOC measurements of each of the 12 isolates and the medium alone (control). Rows represent the different VOCs (green, low abundance; red, high abundance), several of which were putatively annotated (see Supplementary Table S2).

**Figure 5:**
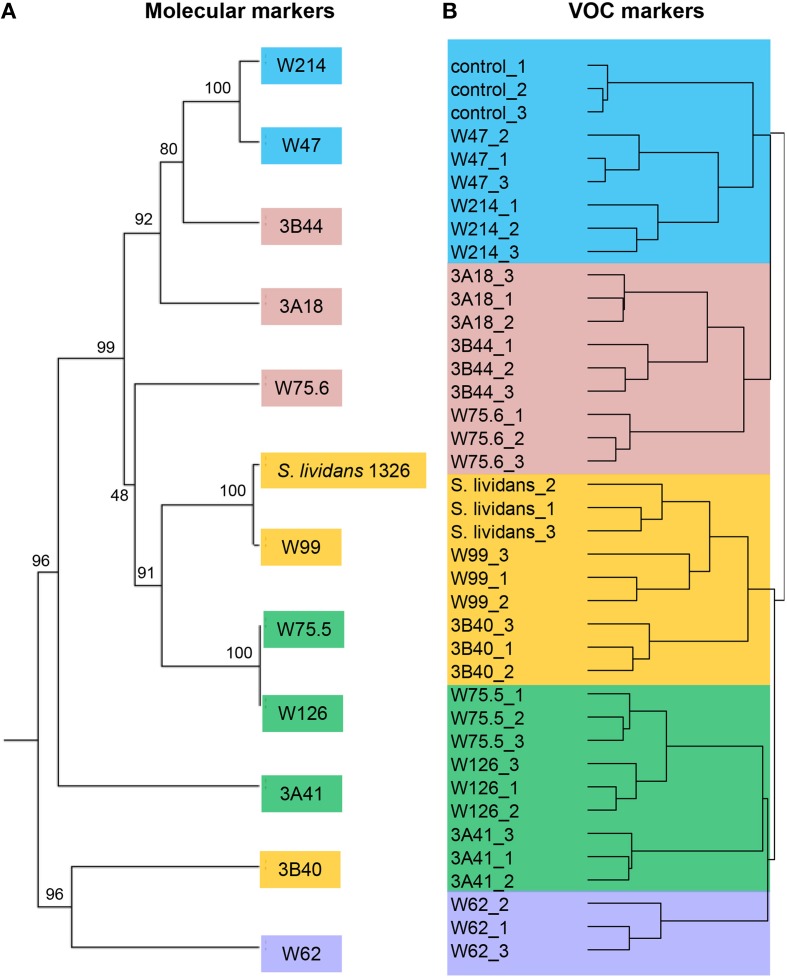
**(A)** Phylogenetic tree of concatenated partial sequences of 16S rRNA, *atpD* and *recA* genes of 11 *Streptomyces* isolates from the *Rhizoctonia*-suppressive soil and the reference strain *S. lividans* 1326. The tree was constructed using UPGMA method and Tamura-3 parameter calculation model with gamma distribution and 1000 bootstrap replicates. **(B)** Hierarchical cluster analysis (HCA) of *Streptomyces* VOCs with UPGMA method and Pearson's correlation coefficient. Different colors indicate different clusters of isolates based on VOC profiles.

For the 12 *Streptomyces* isolates (11 rhizosphere isolates and reference strain *S. lividans* 1326) grown on GA medium and the medium alone (control), a total of 536 VOCs were detected in the headspace. Out of these, 381 VOCs that were significantly different (ANOVA, *p* < 0.05) and detected at intensities at least twice as high as in the control were considered for further analyses. The diversity of VOCs produced by the different *Streptomyces* isolates is shown in Supplementary Table S2 and highlighted in the heat-map (Figure 4). The VOCs detected belong to diverse classes of compounds such as alcohols, aldehydes, carboxylic acids, esters, ketones, sulfur compounds, and several terpenes (Supplementary Table S2). Most VOCs were found to be specific for some *Streptomyces* isolates and 45 VOCs were found to be commonly produced by all isolates tested. Geosmin (trans-1,10-dimethyl-trans-9-decalol, RI 1423; Supplementary Table S2) was one of these common VOCs. HCA of the VOC profiles resulted in a similar clustering of the 12 *Streptomyces* isolates as the clustering based on the different molecular markers (Figure 5). In contrast to the molecular markers, however, VOC profiling allowed differentiation between closely related *Streptomyces* isolates such as *Streptomyces* strains W75.5 and W126 as well as *Streptomyces* strains W47 and W214.

### Effect of *Streptomyces* VOCs on fungal and plant growth

**Figure 6:**
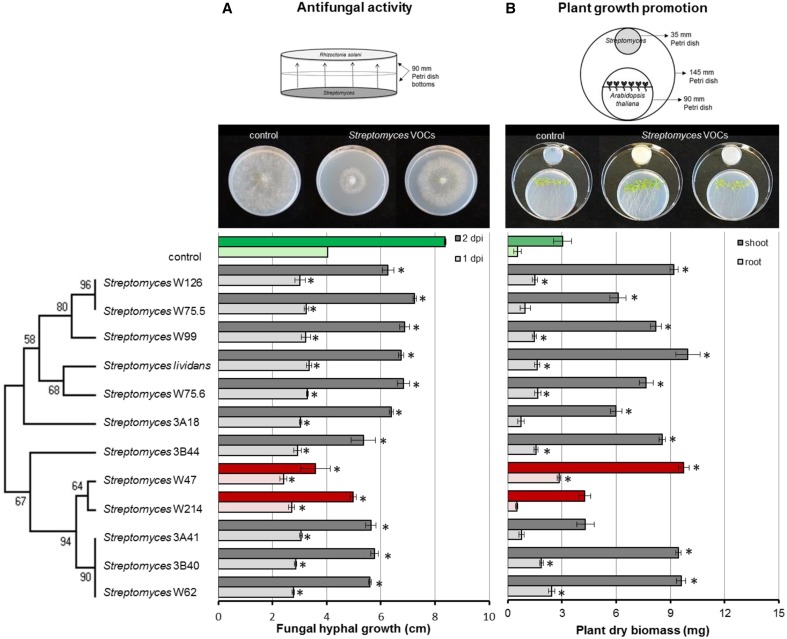
**Inhibition of fungal growth after 1 and 2 days of exposure to ***Streptomyces*** VOCs (A) and growth of ***Arabidopsis thaliana*** seedlings after 2 weeks of exposure to ***Streptomyces*** VOCs (B)**. The controls are displayed in green and isolates with the strongest antifungal activity in red. Bars represent standard errors of the mean of 3 independent biological replicates. Asterisks indicate a statistical difference as compared to controls (exposed to medium only) using Student's *t*-Test (*p* < 0.05, *n* = 3). Pictures of antifungal activity and plant growth promotion were made after 3 and 14 days of exposure, respectively.

To test the antifungal activity of VOCs produced by the *Streptomyces* isolates from disease suppressive soil, hyphal growth of *R. solani* was measured during exposure to VOCs from each of the isolates. In the control, fungal hyphae reached the edge of the agar plates after 2 days of incubation. All *Streptomyces* strains were able to significantly retard the growth of *R. solani*. *Streptomyces* strains W47 and W214 were the most inhibitory. When exposed for 2 days to the VOCs produced by these isolates, radial hyphal growth was reduced by 57 and 41%, respectively (Figure 6A).

Additionally, we tested whether *Streptomyces* VOCs could promote plant growth. To that end, we exposed 7-day-old *A. thaliana* seedlings to VOCs from each of the isolates and determined root and shoot biomass. After 2 weeks of exposure to *Streptomyces* VOCs, no negative effects on plant growth were observed. Ten out of 12 isolates significantly increased shoot biomass, and 8 significantly increased root biomass compared to the control (Figure 6B). *S. lividans* 1326, and *Streptomyces* strains W47 and W62 led to the largest increase in plant biomass, whereas *Streptomyces* strains W214 and 3A41 did not increase shoot and root biomass.

### Identification of *Streptomyces* VOCs contributing to antifungal activity

Since *Streptomyces* strains W47 and W214 are phylogenetically closely related and both showed strong antifungal activity, these isolates were selected to identify VOCs with activity against *R. solani*. Screening of VOCs with potential antifungal activity was computed with One-way ANOVA [*p* < 0.05; with false discovery rate (FDR) correction] and a fold change >2 using the peak intensity of VOCs from W214/control and W47/control. For the selection of VOCs for *in vitro* antifungal activity, three criteria were used: (1) match factor and reverse match factor higher than 850, (2) reliable annotation based on retention indices and, (3) availability of pure (synthetic) reference compounds.

**Figure 7:**
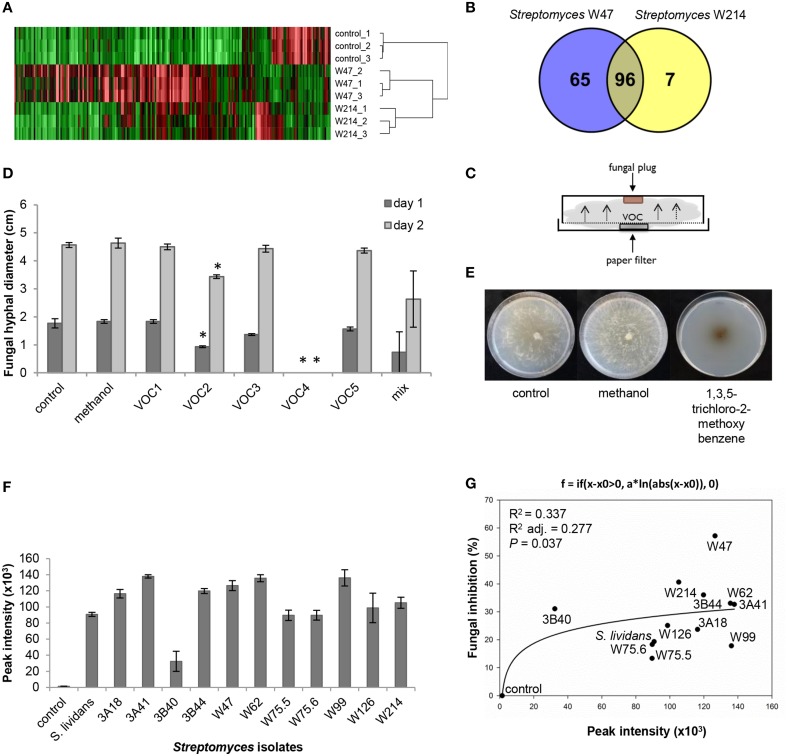
**(A)** VOC profiles of *Streptomyces* strains W47 and W214 compared to control (medium only). **(B)** Venn diagram for common and unique VOCs produced by *Streptomyces* strains W47 and W214. **(C)** Experimental set-up for *in vitro* antifungal activity assay with synthetic VOCs. **(D)** *In vitro* antifungal activity with synthetic VOCs at 1 M [control, methanol, VOC1 (methyl butanoate), VOC2 (methyl 2-methylpentanoate), VOC3 (methyl 3-methylpentanoate), VOC4 (1,3,5-trichloro-2-methoxy benzene), VOC5 (3-octanone)]. Methanol was used to dilute all VOCs. Bars represent standard errors of the mean of 3 independent replicates. Asterisks indicate statistical differences compared to control according to Student's *t*-Test (*p* < 0.05, *n* = 3). **(E)** Fungal growth after exposure to 1,3,5-trichloro-2-methoxy benzene. **(F)** Abundance of 1,3,5-trichloro-2-methoxy benzene produced by different *Streptomyces* isolates based on GC-MS peak intensities. **(G)** Nonlinear relationship between fungal growth inhibition and abundance of 1,3,5-trichloro-2-methoxy benzene.

A comparison of the VOC profiles of *Streptomyces* strains W47 and W214 with the control (medium only) pinpointed VOCs potentially involved in antifungal activity (Figure 7A). A total of 96 VOCs were shared between these two isolates; 65 and 7 VOCs were unique for *Streptomyces* strains W47 and W214, respectively (Figures 7A,B). Since both *Streptomyces* strains W47 and W214 showed antifungal activity, we looked into the VOCs detected for both strains. We selected five common VOCs (methyl butanoate, methyl 2-methylpentanoate, methyl 3-methylpentanoate, 1,3,5-trichloro-2-methoxy benzene, and 3-octanone) which could be reliably annotated based on RI and mass spectral similarity and which were commercially available as authentic reference standards. The identity of these compounds was verified by analyzing pure standards by the GC-MS and comparing their mass spectra and RI with those of the VOCs detected for *Streptomyces* strains W47 and W214. Subsequently, different concentrations of these five VOCs were used to test their inhibitory effect on hyphal growth of *R. solani* (Figure 7C). The VOC 1,3,5-trichloro-2-methoxy benzene completely inhibited radial hyphal growth of *R. solani* at concentrations of 1 M and 100 mM (Figure 7D). Exposure to this VOC led to melanization of *R. solani* hyphae (Figure 7E). The VOC methyl 2-methylpentanoate reduced fungal growth by 47 and 25% after 1 and 2 days of exposure, respectively. Additionally, a mix of the 5 synthetic VOCs, each at a final concentration of 200 mM, inhibited hyphal growth by 58 and 42% after 1 and 2 days of exposure, respectively.

To further determine if the antifungal VOC 1,3,5-trichloro-2-methoxy benzene is typically found for *Streptomyces* isolates that inhibit hyphal growth of *R. solani*, we determined the relative amounts of this VOC produced by each of the 12 *Streptomyces* isolates tested in this study. The results show that production of this VOC is widespread among the 12 *Streptomyces* isolates. Moreover, a positive nonlinear correlation was found between the percentage of hyphal growth inhibition and the abundance (peak intensity) of 1,3,5-trichloro-2-methoxy benzene detected for the 12 isolates (Figures 7F,G).

## Discussion

The production of VOCs by microorganisms is known for several decades. Only recently an increasing number of studies reported on the chemical diversity and possible functions of this group of microbial compounds (Schmidt et al., 2015). In comparison to plant VOCs, knowledge about the natural functions of microbial VOCs is still limited (Bitas et al., 2013). Here we studied the diversity and activities of VOCs produced by different streptomycetes from a *Rhizoctonia*-suppressive soil.

VOC profiling has been extensively used for food flavoring and aroma as well as indicators of fungal growth in buildings and in post-harvest management (Morath et al., 2012). More recently, VOC chemotyping allowed not only to identify species- and strain-specific VOCs but also to study soil microbial activity and shifts in microbial community compositions (McNeal and Herbert, 2009; Müller et al., 2013; Trefz et al., 2013). We showed that VOC profiling can be used for chemotyping different streptomycetes. Most of the 381 VOCs detected for the different streptomycetes from the *Rhizoctonia*-suppressive soil were found to be specific for some isolates whereas fewer VOCs were found to be commonly produced by all isolates. The best known VOCs from streptomycetes are 2-methylisoborneol (MIB) and trans-1,10-dimethyl-trans-9-decalol (geosmin) which are responsible for the characteristic musty or earthy smell of moist soils (Gerber, 1968; Jiang et al., 2007). Our results also show that these VOCs are widely produced by *Streptomyces* isolates from the rhizosphere of sugar beet plants grown in *Rhizoctonia*-suppressive soil. Geosmin was detected for all isolates, whereas MIB was detected for eight isolates. Members of the *Streptomyces* genus differ greatly in their morphology, physiology, and biochemical characteristics (Anderson and Wellington, 2001). Taxonomic delineation of this genus remains complex and leads to over- or under-classified groups. Current approaches for classification of *Streptomyces* as well as other prokaryotes rely on genetic and phenotypic traits, mainly on 16S rRNA gene sequences. This molecular marker, however, is not always sufficient to discriminate between closely related species and between strains of a given species (Girard et al., 2013). We showed that concatenation of *atpD, recA*, and 16S rRNA gene sequences displayed a better phylogenetic delineation of the different streptomycetes than 16S rRNA gene sequences alone, although closely related isolates could not be distinguished. We revealed that VOC profiling allowed discrimination of *Streptomyces* isolates that are phylogenetically close but phenotypically different, such as *Streptomyces* strains W75.5/W126 and W47/W214.

The genus *Streptomyces* is well-known for the production of several antifungal and antiviral compounds and accounts for 80% of the currently available antibiotic compounds (Watve et al., 2001). *Streptomyces* also produces VOCs which reduce the incidence and/or the severity of several plant diseases caused by fungi and cause morphological abnormalities in different fungi (Moore-Landecker and Stotzky, 1973; Wan et al., 2008; Boukaew et al., 2013; Wang et al., 2013; Wu et al., 2015). VOCs produced by the streptomycetes tested here exhibited antifungal and plant growth promoting properties. Several isolates inhibited hyphal growth, with *Streptomyces* strains W47 and W214 showing the strongest inhibitory effect. Given that these streptomycetes were obtained from a *Rhizoctonia*-suppressive soil suggests that VOCs may contribute to disease suppressiveness. This suggestion needs to be further investigated *in situ* but fits well with one of the initial hypotheses of Lockwood (Lockwood, 1977) for the potential role of microbial VOCs in soil fungistasis. To provide more conclusive proof of the role of these *Streptomyces* VOCs in disease suppression in the soil ecosystem, specific soil bioassays are needed where the VOC producers and the pathogen are physically separated. However, there are several technical limitations to accomplish this. First, the strains used here are rhizospheric bacteria that need to be positioned in their ecological context (the rhizosphere) to provide meaningful results. Given the need for the localization of the *Streptomyces* strains in the rhizosphere where also the pathogen colonizes and infects, it has not been possible yet to physically separate the *Streptomyces* strains from the fungal pathogen. This is due in part to the prolific growth of this particular fungus. The physical separation *in situ* is needed to exclude a possible role of mechanisms other than VOCs. An alternative approach would be to generate site-directed mutants of the *Streptomyces* strains that do not produce one or more of the specific VOCs identified in this study. Comparison of the activity of these mutants with their wildtype strains would then more conclusively resolve the role of specific VOCs in disease suppression *in situ*. For this alternative approach, however, we have not yet been able to generate mutants as many environmental *Streptomyces* species/strains are not or very difficult to access for genetic modification.

Several studies have described antifungal activity by bacterial VOCs, however, few have identified single or blends of VOCs responsible for the antifungal activity (Kai et al., 2007; Wang et al., 2013). For *Pseudomonas*, six VOCs (cyclohexanal, decanal, 2-ethyl 1-hexanol, nonanal, benzothiazole, and dimethyl trisulfide) were found to inhibit mycelial growth and sclerotial germination of *Sclerotinia sclerotiorum* at tested volumes of 100 and 150 μl (Fernando et al., 2005). Regarding VOCs produced by *Streptomyces* species, butanone (methyl vinyl ketone) and dimethyl disulfide were described to inhibit the spore germination in *Cladosporium cladosporioides* and mycelial growth of *Fusarium moniliforme*, respectively (Herrington et al., 1987; Wang et al., 2013). Here we showed that two out of five VOCs detected for *Streptomyces* strains W47 and W214 (methyl 2-methylpentanoate and 1,3,5-trichloro-2-methoxy benzene) as well as the mix of these VOCs exhibited antifungal activity, albeit at high concentrations. The VOC 1,3,5-trichloro-2-methoxy benzene completely inhibited fungal growth and caused melanization of the fungal hyphae. 1,3,5-Trichloro-2-methoxy benzene is also known as 2,4,6-trichloroanisole (TCA) and causes off-flavor in wine, coffee and water (Spadone et al., 1990; Jensen et al., 1994). Anisole produced by *S. albulus* has recently been described for activity against *S. sclerotiorum* and *F. oxysporum* (Wu et al., 2015). Derivatives of anisole have been described to be produced by bacteria and fungi (Mauriello et al., 2004; Blom et al., 2011), but no function has been ascribed to this specific VOC yet. To our knowledge, this is the first time that 1,3,5-trichloro-2-methoxy benzene is described for its antifungal activity. The VOC methyl 2-methylpentanoate, which also exhibited antifungal activity, is known for other streptomycetes, but also for this VOC no specific function has been described so far (Wilkins and Scholler, 2009; Dickschat et al., 2011). For both 1,3,5-trichloro-2-methoxy benzene and methyl 2-methylpentanoate, the concentrations needed to inhibit fungal growth were high. However, in the experimental setup used here, we do not know how much of the applied VOCs actually contact the fungal hyphae, which part of the fungal hyphae are the most VOC sensitive and how long VOC exposure is necessary to exert the antifungal activity. These aspects will be subject of future studies. Also, the identification of *Streptomyces* VOCs involved in plant growth promotion was not further pursued in this study but a possible candidate is acetoin (3-hydroxy-2-butanone) which was detected for several isolates tested here. Acetoin and 2,3-butanediol were the first bacterial VOCs described for their role in plant growth promotion (Ryu et al., 2003). More recently, other VOCs have been identified for their role in plant growth promotion such as indole, 1-hexanol, pentadecane, 13-tetradecadien-1-ol, 2-butanone, and 2-methyl-n-1-tridecene (Blom et al., 2011; Park et al., 2015). Plant growth-promoting effects can also be, at least partially, due to CO_2_ accumulation as products of microbial metabolism when using closed Petri dishes (Kai and Piechulla, 2009). In the experimental set-up used in our study, however, CO_2_ appears to have only a minor role since two isolates (3A41 and W214) out of the 12 tested isolates did not promote shoot and root growth, and two isolates (3A18 and W75.5) did not promote root growth.

In conclusion, VOCs produced by rhizosphere-associated streptomycetes are chemically diverse and display antifungal and plant growth-promoting properties. Hence, VOC profiling can provide a new resource of novel metabolites and biochemical pathways involved in antifungal activity and plant growth promotion by streptomycetes. We identified two VOCs with antifungal activity, but it remains to be determined whether these compounds are produced *in situ* at the biologically relevant concentrations. Our work further demonstrated the utility of VOC profiling for the characterization of streptomycetes, providing an additional tool for phylogenetic delineation of closely related strains.

## Author contributions

VC designed and performed the experiments and drafted the manuscript. GV and HZ assisted with the isolation of the Actinobacteria. VJC assisted with the molecular characterization of the *Streptomyces* isolates. VC, RM, and DE analyzed the GC-MS data. JR supervised the work and assisted with the experimental design and writing. All authors revised the manuscript and approved submission.

### Conflict of interest statement

The authors declare that the research was conducted in the absence of any commercial or financial relationships that could be construed as a potential conflict of interest.
