# Supplementary material for: Diversity and functions of volatile organic compounds produced by Streptomyces from a disease-suppressive soil
Source: Front Microbiol. 2015 Oct 9;6:1081. doi: 10.3389/fmicb.2015.01081 (PMC4598592; doi:10.3389/fmicb.2015.01081)
Supplement: Supplementary file 1 [file Table1.PDF]

Supplementary Table S1. Selective treatments and media used for the isolation of Actinobacteria from suppressive soil.

Pre-treatment	Medium	Incubation	Reference
6% yeast extract (30°C, 120 min)	HA, GCA, SFM, MM<br>50 µg/ml nystatin<br>10 µg/ml nalidixic acid	1-3 weeks 30°C	Hayakawa & Nonomura, 1987<br>Hayakawa & Nonomura, 1989<br>Kuester & Williams, 1964<br>Zhang, 1985<br>Kiezer et al., 2000<br>Zhu et al., 2015
none	HV<br>20 µg/ml nalidixic acid<br>20 µg/ml trimethoprim<br>100 µg/ml delvocid*	3 weeks 30°C	Hayakawa et al., 1991<br>Tamura et al. 1997<br>Zhu et al., 2015
rehydration (30°C, 90 min) centrifugation	HV<br>20 µg/ml nalidixic acid<br>20 µg/ml trimethoprim<br>100 µg/ml delvocid*	3 weeks 30°C	Hayakawa et al., 2000
none	CN<br>100 µg/ml delvocid*	2-3 weeks 25-30°C	Gavrish et al., 2010
none	TSAYE<br>100 µg/ml delvocid*	10 days 25°C	Hagedorn and Holt, 1975
1.5% phenol (30°C, 30 min)	HV	3 weeks 30°C	Hayakawa et al., 2004
wet-heating (70°C, 15 min)	HHVA<br>50 µg/ml nystatin<br>20 µg/ml nalidixic acid	4 weeks 25°C	Seong et al., 2001

*Cycloheximide was replaced by delvocid
