# Supplementary material for: Diversity and functions of volatile organic compounds produced by Streptomyces from a disease-suppressive soil
Source: Front Microbiol. 2015 Oct 9;6:1081. doi: 10.3389/fmicb.2015.01081 (PMC4598592; doi:10.3389/fmicb.2015.01081)
Supplement: Supplementary file 2 [file Table2.PDF]

Supplementary Table S2. List of putative VOCs produced by the 11 *Streptomyces* isolated from a *Rhizoctonia*-suppressive soil and the reference strain *S. lividans* 1326. VOCs displayed are significantly different ($p<0.05$), detected at peak intensities at least twice as high as in the control (medium only) and with a match factor higher than 800. Compounds were putatively annotated by comparing their mass spectra with those of NIST and in-house mass spectral libraries.

**RI**	**Putative compound**	**3A18**	**3A41**	**3B40**	**3B44**	**W47**	**W62**	**W75.5**	**W75.6**	**W99**	**W126**	**W214**	***S. lividans***
737	3-Hydroxy-2-butanone	X	X		X	X			X	X		X	
743	Methyl butanoate		X	X	X	X	X		X	X	X	X	X
745	Butanoic acid, methyl ester	X		X	X	X				X			X
749	3-Methyl-3-buten-1-ol	X	X	X	X	X	X	X	X	X	X	X	X
751	1-Butanol, 3-methyl	X	X	X	X	X		X	X	X	X	X	X
754	1-Butanol, 2-methyl-, (S)	X	X	X	X	X		X	X	X	X	X	X
760	Dimethyl disulfide	X	X	X	X	X	X	X	X	X	X	X	X
774	2-Methylpropanoic acid					X			X				
792	Butanoic acid		X	X	X	X			X	X		X	
796	Furan, 2-propyl								X				
799	Butanoic acid, 2-methyl-, methyl ester	X	X	X	X	X	X		X	X	X	X	X
805	meso-3,4-Hexanediol	X	X	X	X	X			X	X		X	X
807	Propanoic acid, 2-hydroxy-2-methyl-, methyl ester	X	X	X	X	X			X	X		X	X
812	Oxirane, (methoxymethyl)	X	X	X	X	X	X		X	X		X	X
817	Acetic acid, butyl ester		X	X			X						X
818	Butanoic acid, 3,3-dimethyl-, methyl ester		X	X	X	X	X		X		X	X	
850	Butanethioic acid, S-methyl ester	X	X	X	X	X	X	X	X	X	X	X	X
856	2-Methylbutanoic acid			X		X			X	X		X	X
858	2-Hexanone, 5-methyl	X	X		X	X	X	X	X	X	X	X	X
861	Butanoic acid, 2-ethyl-, methyl ester			X	X	X			X	X		X	X
867	2-Butenoic acid, 2-methyl-, methyl ester, (E)-		X	X		X	X	X	X	X	X		X
869	Pentanoic acid, 2-methyl-, methyl ester			X	X	X	X	X	X	X	X	X	X
886	1-Propanone, 1-(2-furanyl)							X			X		
888	Butanethioic acid, S-methyl ester	X	X	X	X	X			X		X	X	
890	Pentanoic acid, 4-methyl-, methyl ester			X		X	X	X	X		X		
894	5-Hydroxy-4-octanone	X	X	X	X	X	X		X	X	X	X	X
899	2-Heptanol	X				X			X			X	
921	Pentanoic acid, 2,4-dimethyl-, methyl ester	X	X	X	X	X	X	X	X	X	X	X	X
925	Hexanoic acid, methyl ester			X		X	X	X	X		X	X	X
927	alpha-Phellandrene	X		X	X				X		X		
929	2-Pentenoic acid, 4-methyl-, methyl ester						X				X		
935	alpha-Pinene	X			X	X	X		X				
935	5-Methylhexane-2,4-dione, enol	X	X	X	X	X	X	X	X	X	X	X	X
939	3-Pentenoic acid, 4-methyl-, methyl ester	X	X	X	X	X	X	X	X	X	X	X	
940	S-Methyl 3-methylbutanethioate	X	X	X	X	X	X	X	X	X	X	X	X
946	Pentanoic acid, 4-methyl	X	X	X	X	X		X	X		X	X	X

**RI**	**Putative compound**	**3A18**	**3A41**	**3B40**	**3B44**	**W47**	**W62**	**W75.5**	**W75.6**	**W99**	**W126**	**W214**	***S. lividans***
948	Pentanoic acid, 2-ethyl-, methyl ester			X	X	X	X		X	X		X	X
951	Camphene	X		X	X	X	X		X				
962	Benzaldehyde												X
965	Pentanoic acid, 2,4-dimethyl-, methyl ester	X	X		X	X	X	X	X	X	X	X	X
970	1-Heptanol				X			X					
973	5-Methylhexane-2,4-dione, enol	X	X	X	X	X	X	X	X	X	X	X	X
973	Dimethyl trisulfide	X	X	X	X	X	X	X	X	X	X	X	X
974	2,4-Heptanedione	X	X		X	X		X			X	X	
976	Methyl 2-furoate			X			X						
980	beta-Pinene	X			X		X		X				
982	1,3- Cyclopentadiene, 1,3-bis(1-methylethyl)	X		X	X	X	X	X	X	X	X		X
984	Pentane, 3-bromo							X			X		
987	3-Octanone	X	X		X	X	X	X	X		X	X	
989	2,4- Cycloheptadien-1-one, 2,6,6-trimethyl	X		X	X	X	X		X	X			X
989	Hexanoic acid, 5-methyl-, methyl ester	X	X	X	X	X	X	X	X	X	X	X	X
991	3(2H)-Thiophenone, dihydro-2-methyl							X			X		
998	4-Methylhexanoic acid, methyl ester	X	X	X		X	X	X	X		X	X	
1019	Hexanoic acid, 2,4-dimethyl-, methyl ester, (2DL,4L)	X		X	X	X	X	X	X	X	X	X	X
1023	p-Menth-8-ene, 3-methylene	X	X	X	X	X	X	X	X	X	X	X	X
1026	Methyl heptanoate	X	X	X	X	X	X	X	X	X	X	X	X
1028	p- Cymene								X				
1029	1-Hexanol, 2-ethyl					X							
1032	Limonene	X					X	X	X		X		
1037	1-Heptene, 6-methyl				X			X			X		
1044	Hexanoic acid, 2-ethyl-, methyl ester				X	X			X				X
1044	Cyclohexanol, 3,3,5-trimethyl-, trans						X	X	X		X		
1054	(R)-(-)-4-Methylhexanoic acid	X	X	X	X	X	X	X	X		X	X	X
1055	Thiophene, 2-methyl-5-propyl	X				X		X	X		X		
1061	7-Methyl-1,6-dioxaspiro[4.5]decane			X	X			X		X	X		X
1063	gamma-Terpinene	X		X	X	X	X	X	X	X	X		X
1072	1-Octanol					X		X					
1089	Methyl 6-methyl heptanoate					X						X	
1094	Terpinolene	X		X	X	X	X	X	X	X	X		X
1095	2-Nonanone	X	X		X	X			X				
1101	Benzoic acid, methyl ester		X	X	X	X	X		X	X			X
1102	2-Nonanol					X							
1103	1,6-Octadien-3-ol, 3,7-dimethyl		X		X	X	X				X		
1125	Heptanoic acid, 2,6-dimethyl-, methyl ester			X	X	X	X		X	X	X	X	X
1127	Octanoic acid, methyl ester			X		X	X		X	X		X	
1132	Cyclohexane, 4-methyl-2-methylene-1-(1-methylethylidene)	X		X	X	X	X		X				
1138	1-Octene, 7-methyl				X			X			X		

RI	Putative compound	3A18	3A41	3B40	3B44	W47	W62	W75.5	W75.6	W99	W126	W214	*S. lividans*
1139	4-Nonanone, 8-methyl		X	X	X			X		X	X		X
1142	cis-Ocimene, 8-oxo	X		X	X	X	X		X				
1150	Benzene, 2,4-dimethyl-1-(1-methylethyl)	X					X		X				
1157	6-Nonen-1-ol, (Z)	X	X	X		X		X		X	X	X	
1163	Octanoic acid, 2-methyl-, methyl ester			X	X	X	X		X			X	
1167	1,3- Cyclopentadiene, 1,3-bis(1-methylethyl)	X	X		X	X	X		X				
1184	Benzeneacetic acid, methyl ester	X	X	X	X	X	X	X	X	X	X	X	
1194	2-Methylisoborneol	X		X	X	X	X		X	X			X
1208	Benzene, 2,4-dichloro-1-methoxy	X	X			X		X	X		X		
1210	Benzene, isothiocyanato												X
1221	Benzoic acid, 3-methyl-, methyl ester	X	X	X	X	X	X	X	X	X	X	X	X
1241	11,12,13-Trinor-5-eudesmene	X	X	X	X	X	X	X	X	X	X	X	X
1274	1-Decanol					X					X		
1275	Bicyclo[4.3.0]non-3-ene, 3,4,7-trimethyl	X		X	X	X	X	X	X	X	X	X	X
1286	2,6-Octadienoic acid, 3,7-dimethyl-, methyl ester								X				
1290	Methyl 8-methyl-nonanoate		X	X	X	X	X	X		X	X	X	X
1296	Cogeijerene	X	X	X	X		X	X	X	X	X		X
1297	2-Undecanone	X	X		X	X	X						
1297	2-Bromo-4-chloroanisole	X	X			X		X	X		X		
1314	(E)-Methyl cinnamate			X			X		X	X			
1328	2,6-Octadienoic acid, 3,7-dimethyl-, methyl ester				X	X			X				
1339	Azulene, 1,2,3,3a,4,5,6,7-octahydro-1,4-dimethyl-7-(1-methylethenyl)-, [1R-(1α,3aβ,4α,7β)]-	X	X	X	X	X	X	X	X	X	X		X
1341	Benzene, 1,3,5-trichloro-2-methoxy	X	X	X	X	X	X	X	X	X	X	X	X
1358	Benzoic acid, 3,5-dimethyl-, methyl ester	X	X	X	X	X	X	X	X	X	X	X	
1361	alpha- Cubebene	X	X	X	X	X	X	X	X	X	X	X	X
1367	African-1-ene		X	X	X	X	X		X	X	X	X	X
1372	Tridecane, 3-methyl										X		
1385	alpha-Ylangene	X	X	X	X	X	X	X	X	X	X	X	X
1390	alpha- Copaene	X	X	X	X	X	X	X	X	X	X		X
1393	2-Propenoic acid, 3-phenyl-, methyl ester, (E)-						X				X		
1397	Methyl 8-methyl-decanoate		X	X	X	X	X	X	X	X	X	X	X
1405	beta-Elemene	X	X	X	X	X	X	X	X	X	X	X	X
1409	2-Undecanone, 6,10-dimethyl		X				X		X	X			X
1415	10-Undecenoic acid, methyl ester		X	X		X	X	X	X	X		X	X
1423	Undecanoic acid, 2-methyl-, methyl ester	X		X	X	X	X	X	X	X	X		X
1423	trans-1,10-Dimethyl-trans-9-decalol	X	X	X	X	X	X	X	X	X	X	X	X
1432	alpha- Cedrene	X	X	X	X	X	X	X	X	X	X	X	X
1435	beta-Ylangene	X	X	X	X	X	X	X	X	X	X	X	X
1442	beta- Cedrene	X	X	X	X	X	X	X	X	X	X	X	X
1447	1H-Isoindole-1,3(2H)-dione, 2-phenyl					X	X		X			X	X
1451	beta-Gurjunene	X	X	X	X	X	X	X	X	X	X		X

RI	Putative compound	3A18	3A41	3B40	3B44	W47	W62	W75.5	W75.6	W99	W126	W214	*S. lividans*
1466	4,7-Methanoazulene, 1,2,3,4,5,6,7,8-octahydro-1,4,9,9-tetramethyl-, [1S-(1α,4α,7α)]-	X	X	X	X	X	X	X	X	X	X	X	X
1471	1,2-Benzenediol, O-(4-butylbenzoyl)-O'-(isobutoxycarbonyl)	X		X	X	X	X	X		X	X		X
1474	beta-Selinene	X	X	X	X	X	X	X	X	X	X	X	X
1477	Cadina-1(10),6,8-triene	X	X	X	X	X	X	X	X	X	X		X
1481	Zizaene	X	X	X	X	X	X	X	X	X	X		X
1482	4,11-Epoxy-cis-eudesmane	X	X	X	X	X	X	X	X	X	X	X	X
1489	Methyl 10-methyl-undecanoate	X	X	X	X	X	X	X	X	X	X		X
1491	Naphthalene, 1,2,3,4,4a,5,6,8a-octahydro-7-methyl-4-methylene-1-(1-methylethyl)-, (1α,4aβ,8aα)-	X	X	X	X	X	X	X	X	X	X	X	X
1495	alpha-Muurolene	X		X	X	X	X	X		X	X		X
1499	Germacrene D	X		X	X	X	X	X		X	X		X
1505	beta-Selinene	X	X	X	X	X	X	X	X	X	X	X	X
1510	Naphthalene, 1,2,3,5,6,7,8,8a-octahydro-1,8a-dimethyl-7-(1-methylethenyl)-, [1R-(1α,7β,8aα)]-	X	X	X	X	X	X	X	X	X	X	X	X
1514	Cubeb-11-ene		X			X	X	X		X	X		
1521	1H-Benzocyclohepten-7-ol, 2,3,4,4a,5,6,7,8-octahydro-1,1,4a,7-tetramethyl-, cis	X	X	X	X	X	X	X	X	X	X		X
1523	Benzene, 1-methoxy-2-methyl		X			X					X		
1526	Dodecanoic acid, methyl ester			X						X			
1526	gamma-Bisabolene, (E)	X	X	X	X	X	X	X	X	X	X	X	X
1528	Dihydroagarofuran, trans	X	X	X	X	X	X	X	X	X	X	X	X
1530	4-Epicubebol	X	X	X	X	X	X	X	X	X	X	X	X
1537	delta-Amorphene	X	X	X	X	X	X	X	X	X	X	X	X
1541	Zonarene	X	X	X	X	X	X	X	X	X	X	X	X
1548	Cedrene	X	X	X	X	X	X	X		X	X	X	X
1553	alpha- Cadinene	X	X	X	X	X	X	X	X	X	X		X
1560	alpha- Calacorene	X	X	X	X	X	X	X	X	X	X		X
1563	2-Tetradecanone	X	X	X	X	X	X	X		X	X	X	X
1568	alpha-Agarofuran	X	X	X	X	X	X	X		X	X	X	X
1571	2-Tetradecanone	X	X	X	X	X	X	X	X	X	X	X	X
1592	7- Caryophyllen-6-ol		X	X			X			X	X		
1596	Methyl 10-methyl-dodecanoate	X	X	X	X	X	X	X		X	X		X
1606	2-Naphthalenemethanol, 2,3,4,4a,5,6,7,8-octahydro-α,α,4a,8-tetramethyl-, [2R-(2α,4aβ,8β)]-	X	X	X	X	X	X	X		X	X		X
1646	Cubenol		X				X	X			X	X	X
1658	2-Naphthalenemethanol, decahydro-α,α,4a-trimethyl-8-methylene-, [2R-(2α,4aα,8aβ)]-	X	X	X	X	X	X	X	X	X	X		X
1664	2-Pentadecanone	X	X	X	X	X	X	X	X	X	X	X	X
1682	7-epi-alpha-Selinene	X	X	X			X	X	X	X	X		X
1699	Heptadecane						X						
1699	Phenol, 2,4-di-t-butyl-6-nitro	X		X		X	X	X		X	X		X
1717	2,2,7,7-Tetramethyltricyclo[6.2.1.0(1,6)]undec-4-en-3-one	X			X		X	X	X	X	X	X	X
1890	Acetic acid, methoxy-, methyl ester		X	X						X	X	X	X
1940	Cembrene			X						X			

RI	Putative compound	3A18	3A41	3B40	3B44	W47	W62	W75.5	W75.6	W99	W126	W214	*S. lividans*
2038	Bicyclo[9.3.1]pentadeca-3,7-dien-12-ol, 4,8,12,15,15-pentamethyl-, [1R-(1R*,3E,7E,11R*,12R*)]		X	X	X		X	X		X			X
