# Supplementary material for: Diversity and functions of volatile organic compounds produced by Streptomyces from a disease-suppressive soil
Source: Front Microbiol. 2015 Oct 9;6:1081. doi: 10.3389/fmicb.2015.01081 (PMC4598592; doi:10.3389/fmicb.2015.01081)
Supplement: Supplementary file 3 [file Image1.PDF]

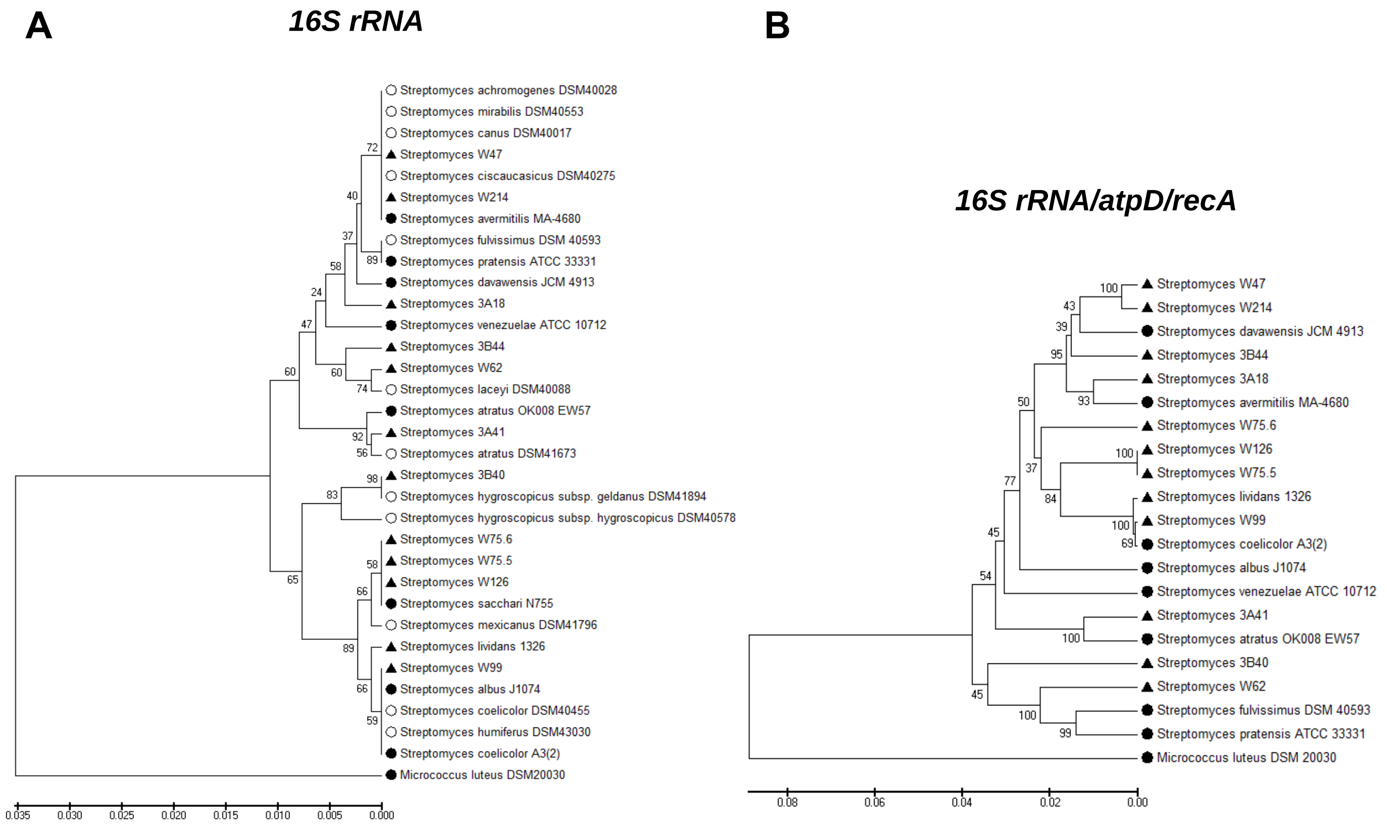

Supplemetary Figure S1. Phylogenetic tree of *Streptomyces* partial sequences of 16S *rRNA* gene **(A)** and concatenated sequences of 16S *rRNA*, *atpD* and *recA* genes **(B).** The tree was constructed using UPGMA method and Tamura-3 parameter calculation model with gamma distribution and 1000 bootstrap replicates. *Micrococcus luteus* DSM200330 was used as the outgroup. ((▲) isolates described in this study, (○) type strains, (●) strains with sequenced genome available on NCBI database).
